# Carbon Dots for Forensic Applications: A Critical Review

**DOI:** 10.3390/nano10081535

**Published:** 2020-08-05

**Authors:** Amy Verhagen, Antonios Kelarakis

**Affiliations:** UCLan Research Centre for Smart Materials, School of Natural Sciences, University of Central Lancashire, Preston PR1 2HE, UK; acaverhagen@uclan.ac.uk

**Keywords:** carbon dots, fluorescence, fingerprinting, anti-counterfeiting, molecular sensing, drugs, explosives

## Abstract

Owing to their superior fluorescence performance, inexpensive synthesis and nontoxic nature, carbon dots (C-dots) are systematically explored in a variety of applications; in this review, we outline and critically discuss recent trends with respect to their potential exploitation in criminal investigation, forensic toxicology and anti-counterfeit interventions. Capitalising on their colour-tuneable behaviour (in the sense that they adopt different colours with respect to the incident radiation), C-dot-based compositions are ideal for the visual enhancement of latent fingerprints, affording improved contrast against multicoloured and patterned backgrounds. As highly sensitive and highly selective optical nanoprobes, C-dots show excellent analytical performance in detecting biological compounds, drugs, explosives, heavy metals and poisonous reactants. In addition, benefiting from their versatile structural and chemical composition, C-dots can be incorporated into ink and polymeric formulations capable of functioning as a new generation of cost-effective barcodes and security nanotags for object authentication and anti-counterfeit applications. Translating these encouraging research outcomes into real-life innovations with significant social and economic impact requires an open, multidisciplinary approach and a close synergy between materials scientists, biologists, forensic investigators and digital engineers.

## 1. Introduction

As one of the most photoactive members of the nanocarbon family, C-dots have paved the way to exciting advances in the interface of materials science, chemistry, nanophysics, biology, medicine and engineering from both a fundamental and an applied point of view. The unique photophysical performance of C-dots stems from their excitation-wavelength-dependent emission coupled with their enhanced resistance to photobleaching [1,2,3]. Their quantum yield largely depends upon their elemental composition (typically C, H, O, N and, in certain cases, heteroatoms such as S and P), their surface functionalisation and the suspension medium [4,5,6]. Pronounced quantum confinement effects have been established for graphene dots, whose cores are composed of a few monolayers of nanosized graphene [7]. Simple oxidative treatments generate carboxyl and carbonyl groups on the C-dots’ surfaces, imparting dispersibility in polar media and securing colloidal stability over a prolonged period of time. In addition, the application of a moderate electrochemical field can induce oxygenated defects as well as modify the degree of conjugation of the carbogenic core [8].

Frequently described as the nontoxic counterparts of quantum dots, C-dots are synthesised inexpensively by means of pyrolysis or hydrothermal processing of abundant natural resources such as agro-waste and biomass [9], grass [10], fruit juice [11], leaves [12], glucose [13], gelatine [14], eggs [15], hair fibres [16], etc. Alternatively, well-defined C-dots can be produced by top-down strategies such as arc discharge, laser ablation, oxidative and electro-oxidative treatment of carbon nanotubes [17], carbon fibres [18], activated carbon [19], exhaust soot [20], etc. In principle, those methods are scalable and rely on simple synthetic protocols followed by standard purification and size exclusion treatments such as dialysis, centrifugation and filtration.

With respect to C-dots’ applications, particular emphasis is given to the development of bioimaging nanoprobes with improved spatial resolution and accuracy [21], nano-vehicles for self-targeting drug delivery [22], photodynamic therapy agents [23], antimicrobial materials [24], advanced sensors for chemical and biological compounds [25], technologies for water and soil decontamination [26], slow-release fertilisers [27], polymer nanocomposites [28], highly efficient photocatalysts [29] and superior energy convertors [30]. 

This review focuses on the application of C-dots within the forensic field and, in particular, with respect to the identification of individuals via intelligent fingerprinting, the anti-counterfeit campaign via the development of tough-to-replicate nanopatterns and molecular detection of biological compounds, illicit drugs, explosives, heavy metals and lethal compounds. These promising advances are discussed critically with a view to identify challenges and opportunities in the field and to encourage interdisciplinary synergies that will support the development of viable and sustainable technologies in the near future. Although several excellent review papers on C-dots have been published recently, to the best of our knowledge, a review focusing on their forensic applications is absent from the literature. In this report, we discuss representative contributions published since 2012 and we provide a critical appraisal on the progress achieved and suggestions for the way forward.

## 2. Discussion 

### 2.1. Structural Characterisation and Fluorescence Behaviour

A number of excellent reviews summarise the sizeable body of work relating to both top-down and bottom-up synthetic strategies used to generate well-defined C-dots, offering an overview of their morphology and remarkable properties [1,2,3,4,5,6,25,26]. Below, we make reference to representative reports in order to briefly introduce the most common characterisation methods used to assess the structural and photophysical characteristics of C-dots.

**Figure 1:**
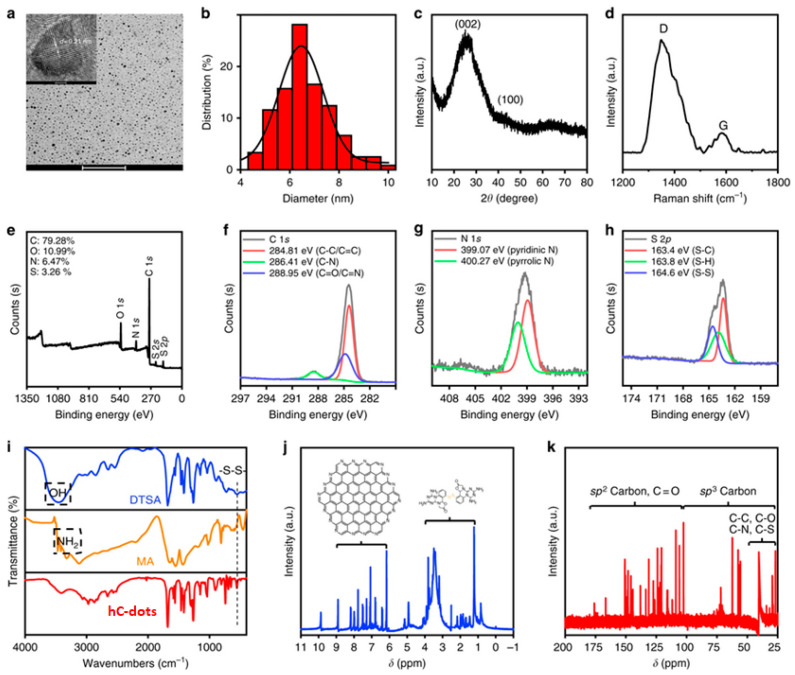
Basic characterisations of hC-dots. (**a**) TEM image of the hC-dots, inset: high-resolution TEM (HR-TEM) image of the hC-dots; (**b**) particles size distribution measured by TEM; (**c**) X-ray diffraction (XRD) pattern of the hC-dots; (**d**) Raman spectrum of the hC-dots; (**e**) XPS spectrum and high-resolution; (**f**) C 1s; (**g**) N 1s; and (**h**) S 2p spectra of the hC-dots; (**i**) FTIR spectrum of DTSA, MA and the hC-dots (the positions marked by dotted rectangles refer to hydroxyl and amino groups; peaks belonging to disulphide bonds are marked by a dotted line); (**j**) ^1^H NMR spectrum (insets: proposed structure of the hC-dot core and surface; the brackets mark out the regions they belong to separately); (**k**) ^13^C NMR spectrum (the brackets mark out the regions related to carbons with different molecular orbitals) of the hC-dots in DMSO-d6. Scale bars: 100 nm (**a**) and 10 nm (**a—inset**). Reproduced from reference [31], Nature Communications; published by Nature Research, 2019.

Yang et al. synthesised hydrophobic C-dots (hC-dots) via the solvothermal treatment of melamine (MA) and a dithiosalicylic acid (DTSA) in acetic acid solution [31]. TEM imaging (Figure 1a) indicates the presence of spherical nanoparticles with narrow size distribution, having an average diameter of 6.5 nm (Figure 1b). The observed lattice spacing of 0.21 nm is consistent with the (100) facet of graphite, pointing to the presence of a graphitic core. The XRD pattern is dominated by a peak centred at approximately 25° and a shoulder at 41°, corresponding to interlayer spacing of 0.34 nm and 0.21 nm, respectively (Figure 1c). The Raman spectrum (Figure 1d) shows two peaks at 1348 cm^−1^ and 1584 cm^−1^ that represent the D and G bands, respectively, while the very high intensity ratio (I_D_/I_G_) indicates the presence of amorphous carbon on the hC-dots’ surface.

The survey XPS spectrum (Figure 1e) displays four peaks, at 284.81, 399.62, 532.22 and 163.89 eV, suggesting the presence of C (79.3%), N (6.5%), O (11%) and S (3.3%), respectively. The XPS spectrum of the C 1s band (Figure 1f) can be deconvoluted into three peaks, at 284.81, 286.41 and 288.95 eV, originating from C–C/C = C, C–N and C = O/C = N, respectively. The N 1s band (Figure 1g) shows two peaks, at 399.07 and 400.27 eV, stemming from pyridinic C_3_–N and pyrrolic C_2_–N–H groups, respectively. The S 2p band in Figure 1h can be deconvoluted into three peaks, at 163.35 eV (S−C), 163.81 eV (S–H) and 164.57 eV (S–S). 

The FTIR spectrum (Figure 1i) indicates the presence of methylene (2876 and 2973 cm^−1^), C ≡ N (2034 cm^−1^), S−H (2650 cm^−1^), amide carbonyl (1682 cm^−1^), C = C (1469 cm^−1^), C−N (1407 cm^−1^), C−S (685 cm^−1^), S−S (491 cm^−1^), aromatic C−NH (1261 cm^−1^) and C−O (1124 cm^−1^) functional groups or chemical bonds. Interestingly, the FTIR spectra of MA and DTSA display peaks related to hydroxyl and amino groups (3064 and 3411 cm^−1^) that are not present in the hC-dots. 

In Figure 1j, the ^1^H NMR spectrum is dominated by peaks at 9.99, 8.3 and 5.75 ppm, attributed to carboxyl protons, protons on the aromatic rings of the graphitised core and NH_2_ protons, respectively. In the ^13^C NMR spectrum, peaks in the range of 30−45 ppm, 100–185 ppm and 170−185 ppm indicate the presence of aliphatic sp^3^ carbon atoms, sp^2^ carbon atoms and carboxyl/amide groups, respectively. 

**Figure 2:**
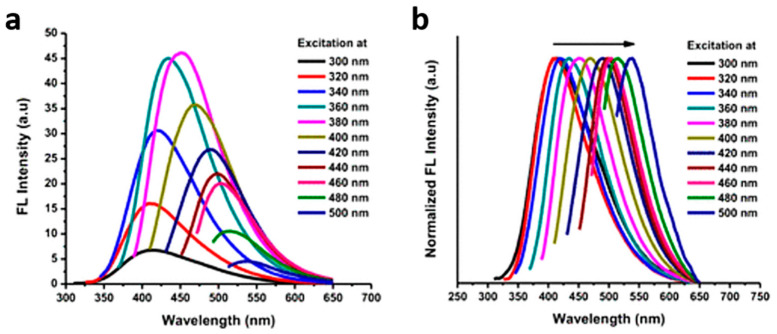
(**a**) Fluorescence spectrum and (**b**) normalised fluorescence spectrum of cranberry-bean-derived C-dots at excitation wavelengths between 300 and 500 nm. Reproduced with permission from reference [34], ACS Omega (https://pubs.acs.org/doi/10.1021/acsomega.9b01333); published by ACS Publishing, 2019. Requests for permission to reuse this material should be directed to the ACS.

In principle, C-dots exhibit characteristic excitation-wavelength-dependent emission, although excitation-wavelength-independent contributions stemming from molecular fluorophores have also been identified [32]. The bandgap transitions of conjugated π-domains and the presence of surface defect states have been recognised as the major factors in the emissive signal [33]. Figure 2a displays the photoluminescence (PL) spectra of cranberry-bean-derived C-dots, while the normalised intensities in Figure 2b emphasise the fact that the emission peaks systematically redshift with increase of the excitation wavelength.

### 2.2. Latent Fingerprint Enhancement

Fingerprint analysis (dactyloscopy) has played a prominent role in criminal investigation for over a century and is also central in identifying victims of disasters, and is by far the most widely used method of biometric identification. The technique is based on the fact that the patterns of epidermal ridges on fingers are unique and characteristic for each individual. Ridge patterns captured electronically or via the “ink and paper” method are stored in national repositories, so that impressions found at crime scenes can be cross-referenced against the police database via an automated process. Today, the rise of high-tech scanners has facilitated fingerprint technologies that are rapidly gaining popularity in everyday applications varying from homeland security to access control and digital authentication for electronic devices.

In terms of latent fingerprint development, early approaches based on silver nitrate, iodine vapour, ninhydrin, cyanoacrylate fuming and vacuum metal deposition have stood the test of time and are still practised in forensics labs. With respect to fingerprint dusting at crime scenes, powders based on aluminium, silica, titania, carbon and magnetic particles have been widely used for decades [35]. Recent advances in nanochemistry can lead to an expanded forensic toolkit by introducing powders and spray formulations based on plasmonic nanoparticles, quantum dots and C-dots that combine strong adhesion to the fingerprint residue, high-resolution imaging and enhanced visibility, while also functioning as molecular recognition agents [36]. For example, gold nanoparticles bearing anti-cotinine antibodies can generate high-quality fingermark impressions while also detecting cotinine (the major metabolite of nicotine) present in the fingermarks, thus providing convincing evidence about the lifestyle habits of the donor [37]. In addition, antibody/magnetic particle conjugates have been successfully applied for the multiplexed detection of drugs of abuse and their metabolites in a single latent fingerprint [38]. 

The working principle for C-dot-based compositions for fingerprint recovery lies in the fact that they adopt different colours when illuminated by different light sources, enabling background-free images and maximising the reliability of fingerprint analysis. While C-dots in the solid state have a tendency to self-quench [39], several strategies have been proposed to overcome this effect, including the use of a diluent matrix [40,41], the development of core–shell nanostructures [42], the incorporation of heteroatom doping [43,44], exploitation of effects such as resonance energy transfer [RET] and π–π interactions [45] and the use of molecular spacers [46].

**Figure 3:**
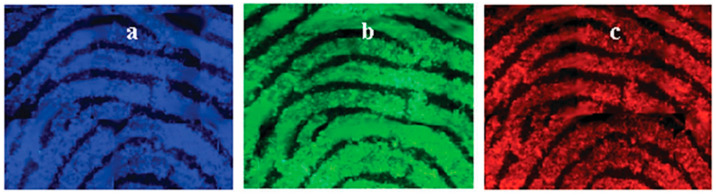
Fluorescence microscopy images of fingerprints deposited on a glass slide and visualised with 0.7% C-dot–silica hybrid nanopowder under (**a**) violet, (**b**) blue and (**c**) green light. The images are composites of smaller images combined using Adobe Photoshop. Reproduced from reference [40] with permission of The Royal Society of Chemistry.

C-dot-based powders for fluorescent visualisation of latent fingerprints were first demonstrated by Fernandes et al. [40], who showed that the incorporation of 0.7 wt. % C-dots into a silica matrix allowed highly detailed and colour-tuneable visualisation of latent fingerprints both on a glass slide and on a multicolour soft drink label. In this study, the C-dots were synthesised via thermal treatment of citric acid monohydrate and ethanolamine, followed by dialysis against water. XPS analysis indicated a composition of C (44.85%), H (5.75%) and N (10.85%), while quantum yield (QY) in water was estimated at 15% under 365 nm excitation using anthracene as a reference. Figure 3 shows the high level of detail revealed fluorescently using the C-dot hybrid powder, as well as the remarkable colour-tuneability accessible via illumination with different excitation wavelengths. This means that, rather than requiring a range of powders to visualise fingerprints against a variety of coloured backgrounds, crime scene investigators can use a single powder alongside a light source capable of producing different wavelengths. Notably, automated fingerprint identification system (AFIS) analysis on a fresh fingerprint developed using the hybrid nanopowder revealed 71 minutiae compared to 65 minutiae revealed from a standard white powder under identical conditions. 

Li et al. [41] incorporated 1% C-dots (synthesised pyrolytically from malic acid and ammonium oxalate) into starch powder and found similarly promising results for the fluorescent visualisation of latent fingerprints on a range of non-porous substrates (glass, white ceramic, black marble, aluminium foil and a coin). In certain cases, the C-dot/starch powder gave better results when compared to 502 cyanoacrylate glue vapour, TiO_2_ powder and iodine vapour. The enhanced fluorescence of this formulation was attributed to favourable interactions of C-dots with the hydroxyl groups in the bulk starch powder.

**Figure 4:**
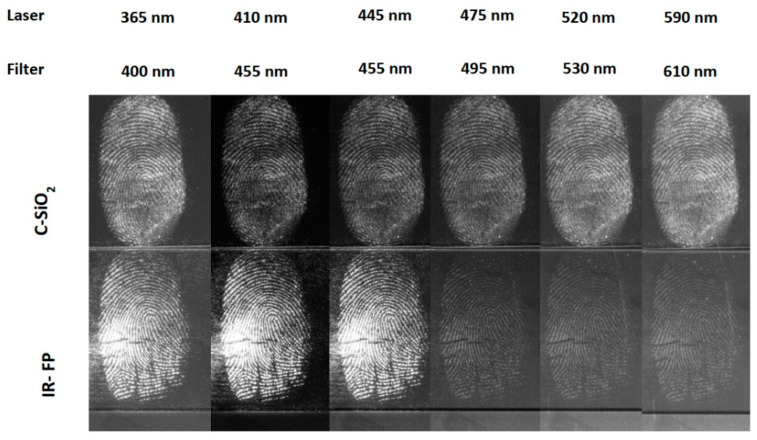
Fingerprints developed with C-SiO_2_ nanopowder (top) and a commercial fluorescent fingerprint powder (bottom) on a glass slide, visualised with a Crime-lite imager. Reproduced from reference [42] with permission of The Royal Society of Chemistry.

Further work by Fernandes and colleagues [42] was focused on carbogenically-coated silica nanoparticles (C-SiO_2_) prepared by treating silica nanoparticles with dimethyloctadecyl [3-(trimethoxysilyl)propyl]ammonium chloride, followed by pyrolysis, surface oxidation with nitric acid, amine functionalisation and, ultimately, dialysis against water. The nanoparticles had an average diameter of 22 nm and contained C (26%), H (4%) and N (5%). The powder showed good flowability, adhered strongly to fingerprints and revealed a higher level of detail (73 minutiae) compared to a commercial white fingerprint powder (65 minutiae) under identical conditions. In addition, the C-SiO_2_ nanopowder effectively revealed high-quality fingerprints, even on strongly fluorescent cardboard, while the commercial white fingerprint powder failed to do so. The C-SiO_2_ nanopowder showed strong contrast when illuminated within the range 365–590 nm, while the commercial fluorescent powder was able to achieve this only under a very limited range of illumination wavelengths (Figure 4). 

Wang et al. [43] reported that nitrogen- and sulphur-doped C-dots (N,S C-dots) can be used as colour-tuneable dusting powders capable of revealing high levels of detail in latent fingerprints deposited on printing paper, plastic, aluminium foil, glass, ceramic and steel, and were equally effective when applied to a thirty-day-old fingerprint. The N,S C-dots were prepared by means of a microwave-assisted method using L-glutathione and citric acid as the precursors, and the resulting material was subjected to centrifugation and dialysis. TEM showed a size distribution of 2–7 nm, while FTIR and XPS confirmed the presence of oxygen-, nitrogen- and sulphur-containing surface functional groups including C = O, OH, NH_2_ and SH, which evidently contributed to their high QY = 48.1% (in 0.1 M H_2_SO_4_ using quinine sulphate as a reference). 

Milenkovic et al. [44] reported that N-doped C-dots (N,C-dots) derived hydrothermally from polyvinylpyrrolidone possess slightly negative charges due to the presence of OH and COOH and can thus attach electrostatically to the proteins contained in fingerprints. AFIS analysis of a fingerprint deposited on a polished metal surface (tweezers) and developed using N,C-dots revealed impressions of superior quality.

Wang et al. [45] employed microwave-assisted pyrolysis of piperazine and phthalic acid to generate graphitic C-dots (pC-dots) with an average size of 1.5 nm. Under 365 nm illumination, the pC-dots adopt a strongly fluorescent yellow-green colour and have a QY of 20.5% in the solid state. These remarkable solid-state fluorescence properties were attributed to RET and direct π–π interactions. The pC-dots were shown to be effective as dusting powders when assessed on fingerprints found on glass, tin foil, plastic, weighing paper, a desk surface, a bottlecap and a coin. 

Jiang et al. [46] reported the synthesis of white-emitting C-dots (wC-dots) by means of thermal treatment of polyoxyethylene sorbitan monooleate dispersed in concentrated phosphoric acid and concentrated sulphuric acid, followed by filtration. The size of the wC-dots was within the range 3.5 to 5.3 nm, while TEM, XRD and Raman spectroscopy confirmed the highly graphitic nature of the carbogenic cores. The presence of long alkyl chains on the surface of the wC-dots played a dual role in effectively suppressing aggregation-induced quenching and enhancing the interactions of the nanoparticles with lipophilic residues present in latent fingerprints. These effects might be directly relevant to the excellent performance of wC-dots as dusting fingerprint powders.

#### 2.2.1. Liquid Formulations

**Figure 5:**
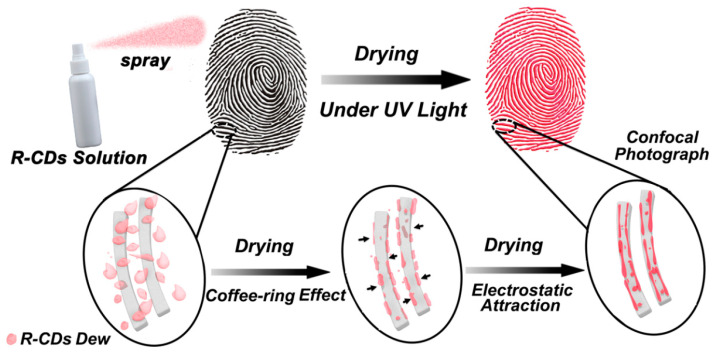
The fingerprint staining process and the mechanism by which the C-dots adhere to the fingerprints. Reprinted with permission from reference [47]. Copyright (2017) American Chemical Society.

The colloidal stability of C-dot dispersions in a variety of liquid media suggests that C-dot-based sprays and other liquid formulations are, in principle, suitable for the visualisation of latent fingerprints, and indeed this has been confirmed in a number of studies. Chen et al. [47] demonstrated that excitation-independent, red-emissive C-dots (rC-dots), with QY = 11.2% in aqueous suspension as measured using an integrating sphere, can be synthesised by hydrothermal treatment of p-phenylenediamine and phosphoric acid. The graphitic rC-dots, with an average diameter of 2.4 nm, were dissolved in diluted hydrochloric acid and applied to latent fingerprints using a spray bottle. The persistent red fluorescence of the sprayed fingerprints was visible in daylight and did not suffer from self-quenching during drying, presumably due to the action of the coffee-ring effect (Figure 5). The strongly fluorescent sprayed impressions revealed a high level of detail, even in fingerprints deposited on challenging substrates such as aluminium foil, leather and plastic. 

**Figure 6:**
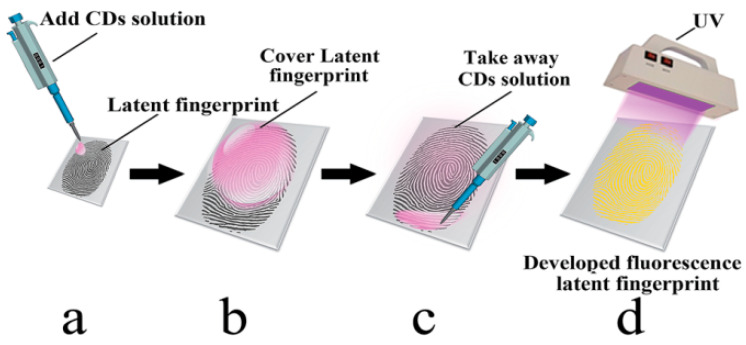
The fingerprint development process: the C-dot solution is added to the fingerprint (**a**), covering it completely (**b**); the solution is then removed by pipette (**c**) and visualised under UV light (**d**). Reproduced from reference [48] with permission from The Royal Society of Chemistry.

Tang et al. [48] reported that excitation-independent, orange-emissive C-dots (oC-dots) with QY = 46% (in water using quinine sulphate as a reference) can be prepared by hydrothermal treatment of rhodamine B, yielding graphitic nanoparticles with average size of 2.1 nm. The oC-dots were dispersed in water and the dispersion was pipetted onto the latent fingerprint and left for 10 s, after which it was removed and the fingerprint was visualised under UV light (Figure 6). This developing process gave rise to high-quality fingerprint impressions, even on multicoloured and highly fluorescent backgrounds. Interestingly, this method was effective for fingerprints up to 120 days old, presumably because they dehydrate as they age and become richer in components like amino acids and sebum that have strong affinity with the oC-dots.

#### 2.2.2. Ink Fingerprinting

Earlier studies have shown that water-based C-dot formulations are ideal for ink fingerprinting, in which the donor’s fingertips are first pressed against the ink pad so that the impressions can be captured on a piece of paper. For example, Su et al. [49] reported that C-dots prepared via microwave treatment of equal weights of urea and citric acid (3 g each), with diameters of 1–5 nm, showed minimal toxicity in beansprouts and mice. Likewise, Xu et al. [50] reported that N-doped C-dots prepared hydrothermally from calcium citrate and urea also showed low toxicity against beansprouts. The aqueous C-dots dispersion reported in the above studies [49,50] were successfully applied for ink fingerprinting.

### 2.3. Anti-counterfeit

The spread of fake goods is associated with designer clothes, watches, sunglasses, cosmetics, drugs, food, oil, tobacco, electrical appliances and electronics, while forgery is frequently encountered in banknotes and documents. Counterfeiting and forgery constitute global crimes that pose serious health and safety risks to consumers, as illicit and substandard drugs not only fail to cure but are responsible for the deaths of thousands of people. This illegal practice and worrying consumer culture deprives bona fide companies of valuable resources and taxable income and undermines national economies. Moreover, infringement of intellectual property through the production of unauthorised replicas discourages investment in innovation and scientific research [51,52]. 

It is therefore crucial for modern societies to implement effective anti-counterfeit and brand protection policies. Mainstream strategies rely on integrated safety features and security graphics such as holograms, optically variable devices, watermarks, laser codes and chemical and biological taggants [53,54]. Unfortunately, even the most sophisticated designs are eventually expertly copied and reproduced. Recent trends highlight the use of polyaromatic organic dyes and quantum dots in security graphics [55]; however, those compounds are prohibitively expensive, cause toxic effects and largely depend on solvent-intensive synthetic routes. 

**Figure 7:**
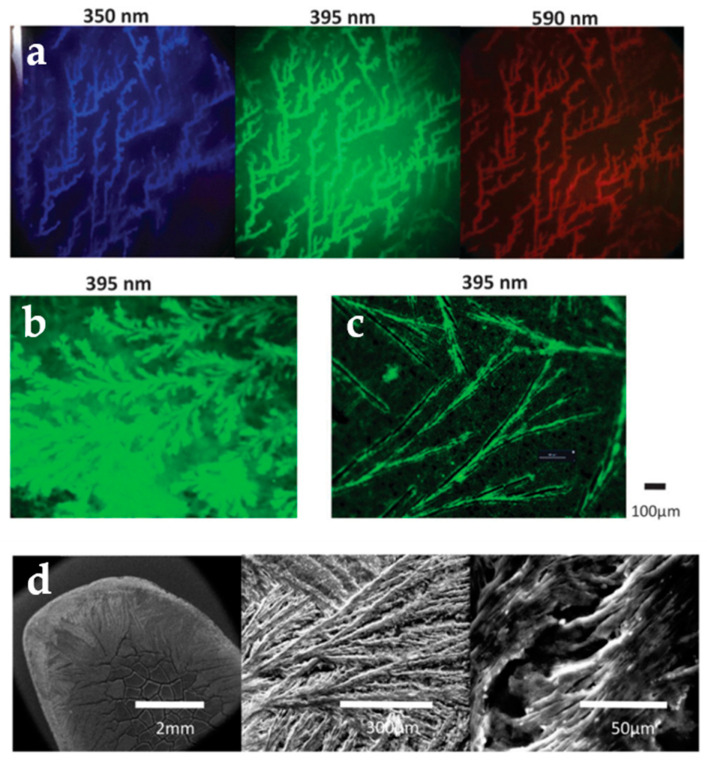
Fluorescence microscopy images of nanotags: (**a**) on glass slides; (**b**) on glass slide at higher concentration; (**c**) on polymer surface; (**d**) under SEM at different magnifications. The excitation wavelengths are indicated. Reproduced from reference [42] with permission of The Royal Society of Chemistry.

To overcome those challenges, innovative and sustainable nanotechnologies based on C-dots have been suggested to support anti-counterfeiting efforts. The working principle for security nano-barcodes based on C-dots is based on their unique optical properties, which can give rise to a potentially unlimited range of unclonable motifs and prints that are nearly impossible to decode and reverse-engineer. Fernandes et al. [42] demonstrated that their previously mentioned C-SiO_2_ nanoparticles lost colloidal stability when the pH of their medium dropped below 8.5, thereafter undergoing spontaneous aggregation as their aqueous medium evaporated and generating complex superstructures (Figure 7) in a nondeterministic manner. These structures would be impossible to duplicate and are therefore ideal for identification and authentication labelling [56]. In principle, the nanotags can be conveniently scanned using a smart phone (equipped with a magnifier and a laser pointer) and can be referenced to a central database for validation. 

**Figure 8:**
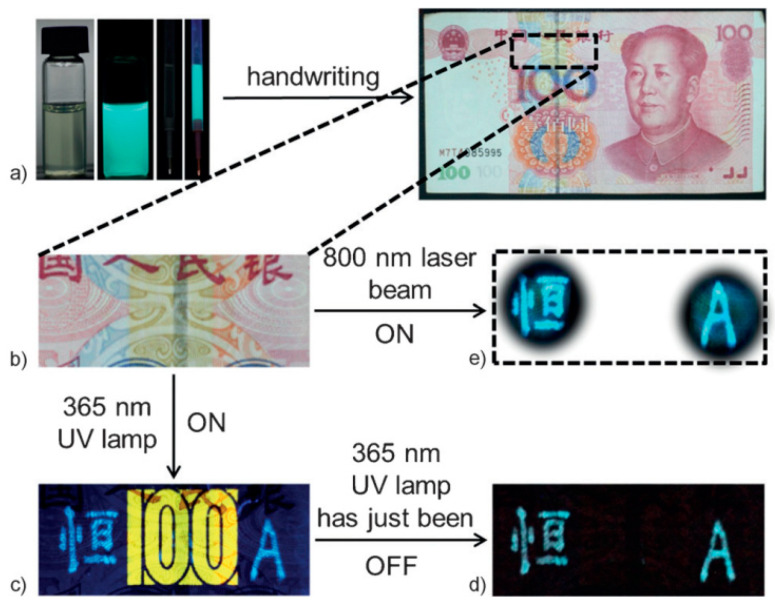
Triple emission mode of C-dots dispersed in a polyvinyl alcohol (PVA) matrix applied in a banknote (**a**,**b**), revealing fluorescence (**c**), room-temperature phosphorescence (RTP) (**d**) and up-conversion photoluminescence (UCPL) (**e**). Reproduced with permission from reference [57], Angewandte Chemie International Edition; Copyright 2016 Wiley-VCH Verlag Gmbh & Co, Weinheim, Germany.

Jiang et al. [57] developed mC-dot dispersions within a polyvinyl alcohol (PVA) matrix that combined photoluminescence, up-conversion photoluminescence (UCPL) and room-temperature phosphorescence (RTP) into a single anti-counterfeit system (Figure 8). The mC-dots were made by dissolving m-phenylenediamine in ethanol and heating the solution in an autoclave for several hours, followed by purification on a silica chromatography column. Both FTIR and XPS confirmed the presence of C = N, C–O and aromatic C–NH_2_ groups, while XPS showed that nitrogen was present in aminic, pyridinic and pyrrolic forms. The RTP excitation spectrum of the mC-dot/PVA composite showed a major band at 360 nm, suggesting that RTP is chiefly due to C–N/C = N bonds, which absorb in this region. The immobilisation of the mC-dots via hydrogen bonding to hydroxyl groups in the PVA matrix is believed to prevent vibrational relaxation of the triplet state induced by UV light, leaving RTP as the only available relaxation mechanism. As illustrated in Figure 8, where a Chinese character and letter ‘A’ are drawn alongside the existing fluorescent anti-counterfeit marking (the number 100) on a Chinese banknote (Figure 8b), the anti-counterfeit system comprises three aspects: firstly, standard fluorescence of both the original marking and the C-dot-drawn markings is revealed under 365 nm light (Figure 8c); when the 365 nm light source is switched off, the original marking disappears immediately, but the C-dot markings remain briefly visible to the naked eye due to RTP (Figure 8d); finally, excitation by an 800 nm laser reveals cyan-coloured, rather than blue-coloured, markings due to UCPL (Figure 8e).

**Figure 9:**
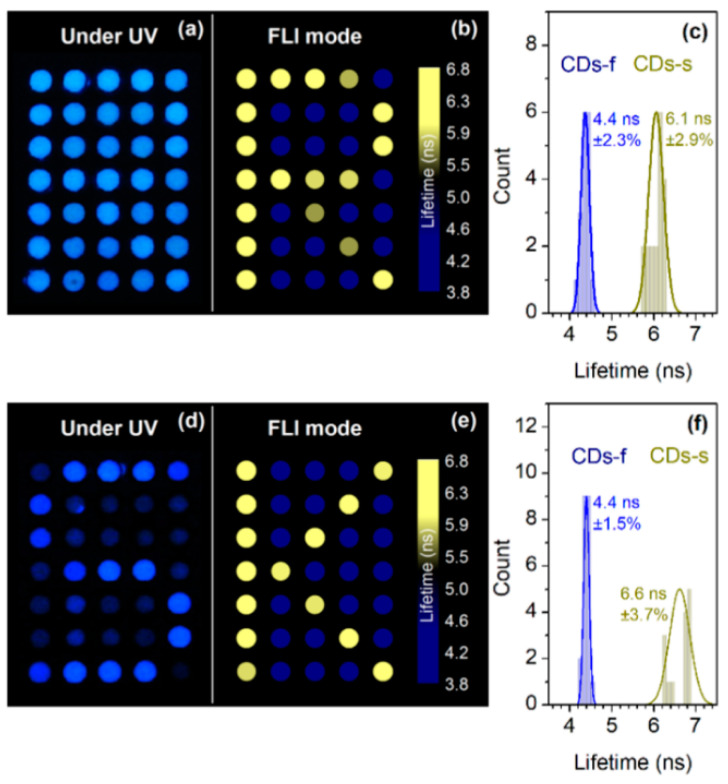
Images printed with C-dot inks and viewed under fluorescence imaging (**a**,**d**) and fluorescence lifetime imaging (**b**,**e**). In (**a**), all pixels have the same concentration of C-dots, while in (**d**), the concentration varies. (**b**) and (**e**) show the clear differentiation in fluorescence lifetime between CDs-f and CDs-s, irrespective of C-dot concentration. Histograms (**c**) and (**f**) show clear separation of fluorescence lifetimes with narrow distributions and no observable overlap. Reproduced with permission from reference [58], ACS Applied Materials and Interfaces; published by ACS Publishing, 2018.

Kalytchuk et al. [58] synthesised C-dots by dissolving citric acid and ethylenediamine in a stainless steel autoclave for 5 h at 200 °C and 220 °C to generate fC-dots and sC-dots, exhibiting “fast” and “slow” fluorescence lifetimes, respectively. TEM analysis showed average diameters of 4.7 and 5.1 nm for fC-dots and sC-dots, respectively. FTIR and XPS indicated that both types of C-dots contain C–C, C = C, C–OH, C = O, C–N and N–H bonds, with the higher synthesis temperature (sC-dots) leading to a higher carbon and a lower oxygen content. At the same time, f-C-dots and sC-dots showed virtually identical UV absorption and emission properties, differing only (and significantly) in their fluorescence lifetimes, which were estimated to be 7.9 and 13.2 ns, respectively. The anti-counterfeiting application of the C-dots is shown in Figure 9: the symbol ‘R’ was encrypted on a paper surface containing 7 × 5 pixels, in which each pixel was printed in ink containing equal concentrations of either fC-dots or sC-dots. Under UV light, all pixels emitted at identical levels and the symbol ‘R’ could not be discerned (Figure 9a). However, using fluorescence lifetime imaging, the symbol ‘R’ can be conveniently decoded (Figure 9b) due to the very different fluorescence lifetimes of the two inks (Figure 9c). When ink formulations with different concentrations of sC-dots and fC-dots are used, the misleading symbol, ‘S’, can be decoded by UV illumination (Figure 9d), while the true encryption symbol, ‘K’, becomes apparent only via fluorescence lifetime imaging.

**Figure 10:**
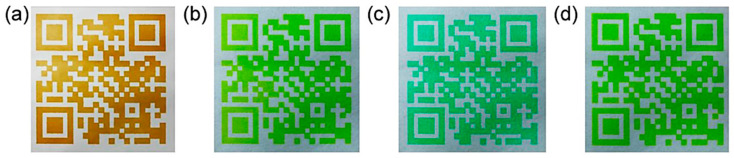
QR code printed with C-dot-based ink and seen under (**a**) natural light and (**b**–**d**) 365 nm UV light; (**c**) is coated with dilute NaOH solution, while (**d**) is coated with dilute acetic acid. Reproduced with permission from reference [59], Carbon; published by Elsevier Ltd., 2019.

Yang et al. [59] reported the synthesis of phosphorus-doped C-dots (P,C-dots) by microwave-assisted pyrolysis of xylose and m-phenylenediamine (dissolved in water) along with H_3_PO_4_, followed by filtration and dialysis. The graphitic P,C-dots were found to have an average diameter of 6.8 nm, while FTIR analysis showed the presence of P–O–(aromatic group) and P–O–H bonds and XPS analysis confirmed the presence of C–N = C, N–C_3_ and N–H bonds. The P,C-dots had QY = 73.6% (in ethanol relative to rhodamine) and when illuminated at 365 nm emitted green light at pH 3-7, while at higher pH, they emitted a blue/green colour. Likewise, a QR code printed with an aqueous dispersion of the P,C-dots appeared golden yellow under natural light (Figure 10a), but under 365 nm radiation, the emission could be adjusted reversibly from green (Figure 10b) to cyan by brushing with dilute NaOH solution (Figure 10c), and back to cyan by brushing with dilute CH_3_COOH (Figure 10d).

**Figure 11:**
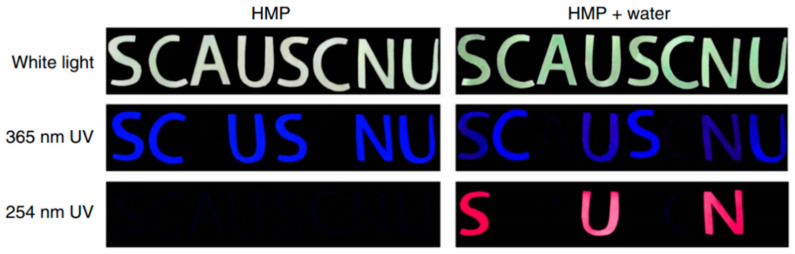
Proof-of-concept security feature made by applying hydrophobic C-dots via marker pen, with and without the addition of water and illuminated under different kinds of light. Reproduced from reference [31], Nature Communications; published by Nature Research, 2019.

Yang et al. [31] reported hydrophobic C-dots (hC-dots) which switch from blue to red fluorescence on addition of water. Their structural characteristics are described in Section 2.1. While well-dispersed hC-dots fluoresce blue under 365 nm illumination, as water is added they begin to aggregate, leading to quenching of the blue fluorescence due to π–π stacking and the emergence of red fluorescence under 254 nm radiation (Figure 11). This reversible two-switch PL behaviour can been exploited in dual-encryption anti-counterfeiting systems such as the one displayed in Figure 11: some letters are printed in a standard, non-fluorescent ink, while the letters ‘SC’, ‘US’ and ‘NU’ are printed using hC-dot ink and the letters ‘C’, ‘S’ and ‘U’ are covered with wax to prevent ingress of water, which would change their fluorescence from blue to red. Under 365 nm illumination, a misleading blue fluorescent code appears, while under 254 nm radiation, the true anti-counterfeit markings are revealed when water is added compared. No markings appear under 254 nm light in the absence of water.

**Figure 12:**
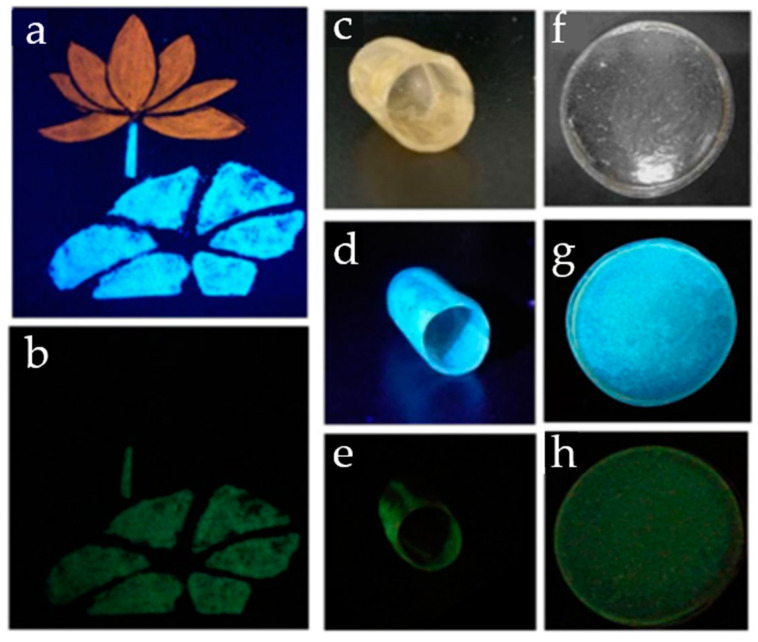
(**a**,**b**): Security feature of a flower printed with fluorescent dye, with its stem and leaf printed with graphene quantum dots–layered double hydroxide (GQD–LDH) nanocomposite (**a**) under 365 nm light and (**b**) after the UV light was switched off. (**c**–**h**): Gelatine capsule (**c**–**e**) and PVA film (**f**–**h**) containing GQD–LDH composite; (**c**,**f**) under natural light; (**d**,**g**) under 365 nm light; and (**e**,**h**) after the UV light was switched off. Reproduced with permission from reference [60], Nano Research; published by Springer Nature, 2018.

Bai et al. [60] developed single-layer graphene quantum dots (GQDs) with average size of 2.7 nm, confined between layered double hydroxide (LDH) layers. The GQD–LDH nanocomposite was prepared via intercalation of the precursor, ethylene diamine tetraactetic acid, into LDH through coprecipitation, followed by calcination. The presence of O- and N-containing functionalities on the surface of GQDs imparts strong interactions with the LDH host. The GQD–LDH nanocomposite exhibits both fluorescence and RTP, showing promising anti-counterfeiting potential for food and drugs as well as documents, bank notes, etc. In an interesting demonstration, the GQD–LDH composite was used alongside a fluorescent dye to create a flower pattern, and separately was incorporated into gelatine capsules and PVA film, giving rise to patterns displaying both fluorescence under UV light and RTP once the UV light was removed (Figure 12). PVA is a widely used and environmentally friendly packaging material, and Liu et al. [61] suggested that incorporation of C-dots into PVA packaging material can be a convenient route to anti-counterfeiting.

Zhu et al. [39] demonstrated the stability of C-dot-based fluorescent inks as well as C-dot-polymer composites used to create random fluorescent patterns for anti-counterfeiting purposes. Both the printed patterns and the C-dot-polymer composite patterns were found to be stable four months after preparation, while the photoluminescence of both was undiminished after 30 min of exposure to 2 kW UV light. Finally, Sk et al. [62] demonstrated that C-dot-based fluorescent ink continued to be visualisable on banknotes even after washing with water followed by washing with soap solution.

### 2.4. Molecular Sensing

Lab-on-a-chip systems are advanced analytical tools that largely rely on integrated microfluidic assays and nanoprobes to achieve minimal processing time, simultaneous detection of several analytes, multiplexed analysis, extreme sensitivity and low sample quantities [63]. Ideally, those portable devices do not require specific skills and extensive training to use, since they are compact and user-friendly with simple functional requirements. Nanosensors bear recognition units which are able to capture molecules of interest, and this process triggers a clear signal, typically optical, electric, mechanical or acoustic in origin [64]. Nanosensors are not only widely considered to be the future of medical diagnostics, being able to recognise underlying health conditions before any symptoms appear, but are equally promising in other fields such as forensic toxicology and trace detection of explosives.

To that end, quantum dots [65,66], plasmonic nanoparticles [67,68,69,70], carbon nanotubes [71] and graphene [72] have been widely explored for the development of affinity-based nanoprobes to detect common narcotics [65,66], anabolic steroids [71], drugs with abuse potential [69], explosives [67,72] and pathogens that can potentially be linked to bioterrorism [68], as well as to determine time since death [70]. Moreover, wearable biosensors can monitor continuously and in real time adverse lifestyle habits associated with criminal behaviour, such as the excessive use of alcohol [73]. 

Coming back to C-dots, their exploitation in forensic detection relates to the development of affinity sensors that bind to certain compounds in a highly selective and highly sensitive fashion. The most common sensing mechanisms of C-dots have been attributed to photo-induced electron transfer (PET), photo-induced charge transfer (PCT), resonance energy transfer (RET) and inner filter effects (IFE) [74]. It suffices to say that identification of illicit drugs and explosives are among the top priorities in law enforcement, while the detection of biofluids encountered in crime scenes and their DNA profiles can play a pivotal role in forensic investigation. Likewise, in a forensic context, acute or chronic exposure to toxic metals and pesticides might be associated with a homicidal or suicidal attempt. 

#### 2.4.1. Detection of Biological Compounds

**Figure 13:**
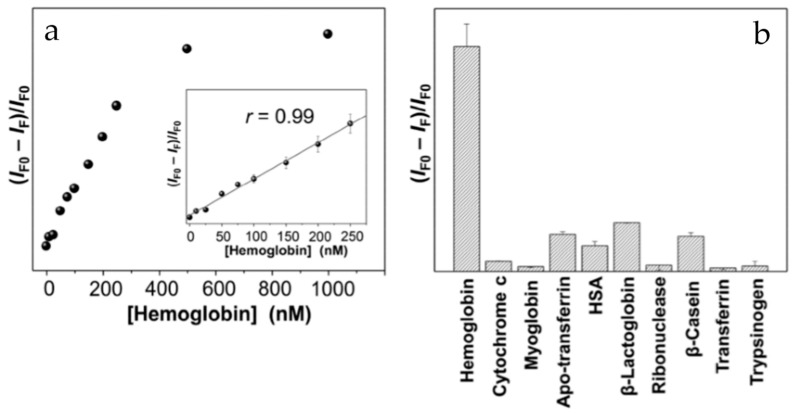
(**a**) Fluorescence quenching in the presence of haemoglobin; (**b**) selectivity of the C-dots for haemoglobin compared to a range of other biological compounds. Reproduced from reference [75] with permission from The Royal Society of Chemistry.

Wang et al. [75] synthesised graphitic C-dots (gC-dots) derived electrochemically from glycine, having a narrow size distribution around 2.4 nm with surface carboxylate and ammonium groups. Addition of haemoglobin within the concentration range 0.05-250 nM led to a linear decrease in the fluorescence intensity (Figure 13a, with minimal interference from related molecules (Figure 13b). The gC-dot system was also successfully used to quantify haemoglobin levels in blood samples.

**Figure 14:**
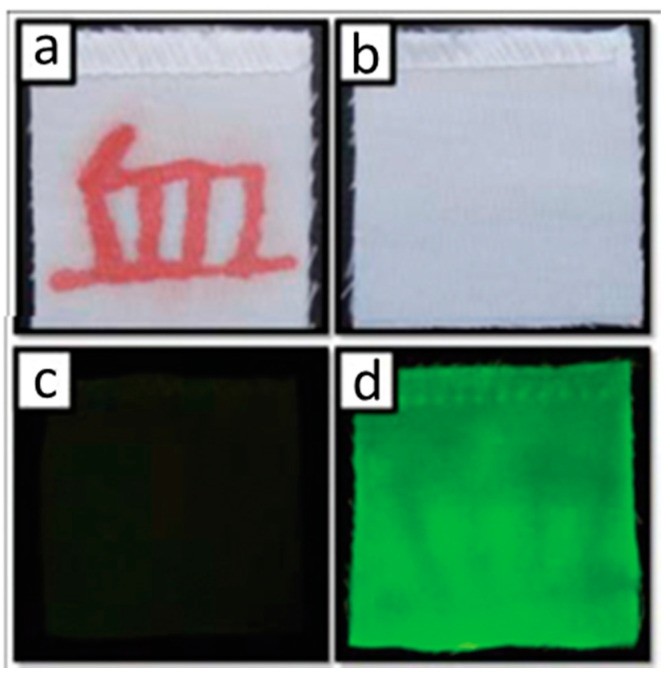
(**a**) Chinese character written in blood; (**b**) the bloodstained fabric after washing; (**c**) the washed fabric sprayed with C-dots and seen under natural light; (**d**) the C-dot-sprayed fabric seen under 460–490 nm light, showing darkening where traces of blood remain. Reproduced from reference [75] with permission from The Royal Society of Chemistry.

To assess the functionality of the gC-dots in a forensic context, a Chinese character was inscribed in blood on a piece of cloth (Figure 14a), which was then washed, rendering the Chinese character invisible (Figure 14b). When sprayed with the gC-dots (Figure 14c) and illuminated under 460–490 nm light, the area which had been bloodstained showed visibly lower fluorescence than the rest of the fabric (Figure 14d). The experiment was repeated with the proteins shown in Figure 13b, as well as with oily pen ink, ballpoint pen ink, soy sauce, ketchup and egg; none of these substances affected the fluorescence of the applied gC-dots, further supporting the high selectivity of this haemoglobin sensor.

**Figure 15:**
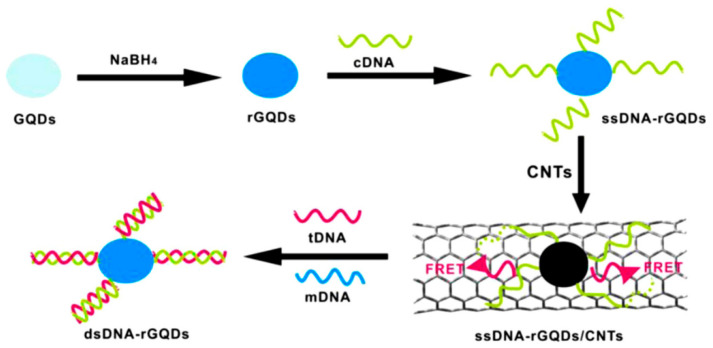
Schematic illustration of the DNA detection principle. Reproduced with permission from reference [76], Biosensors and Bioelectronics; published by Elsevier B. V., 2014.

Qian et al. [76] developed a C-dot/carbon nanotube (CNT) DNA nanosensor that can accurately detect single DNA strands 21 base pairs long. The graphitic C-dots, whose sizes ranged from 2 to 5 nm, were made from graphite which was oxidised by nitric and sulphuric acids, followed by reduction with NaBH_4_ to induce OH and COOH functional groups and enhance the QY. The CNTs, with diameters less than 8 nm, were oxidised with concentrated nitric and sulphuric acids, a process that decreased their length and rendered them water-dispersible. The single-stranded DNA (ss-DNA) was modified with a 5′ terminal amino group which was covalently bonded to the C-dot surface via a condensation reaction. The principle of the detection system is shown in Figure 15: C-dots functionalised with ssDNA, upon the introduction of CNTs, adsorb onto the CNT surface, which quenches their fluorescence through RET. However, upon introduction of the target ssDNA strand (tDNA), which is complementary to the strand attached to the C-dots, the C-dot-bound ssDNA base-pairs with the target strand and is released from the CNT, restoring the C-dots’ fluorescence.

**Figure 16:**
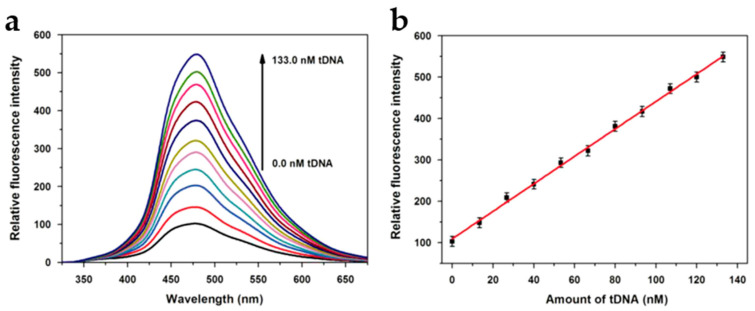
(**a**) Fluorescence recovery after addition of varying concentrations of tDNA; (**b**) linear relationship between fluorescence recovery and tDNA concentration. Reproduced with permission from reference [76], Biosensors and Bioelectronics; published by Elsevier B. V., 2014.

A linear relationship exists between the restored fluorescence intensity and tDNA concentration over the range 1.5–133.0 nM, leading to a detection limit of the system close to 0.4 nM (Figure 16).

Pramanik et al. [77] reported a base-pair-selective DNA sensing system in which C-dots’ fluorescence increases significantly in the presence of double-stranded DNA rich in AT pairs compared to DNA with more CG pairs, with a linear relationship between the fluorescence intensity and the AT percentage. The authors suggest the possibility of using this system in place of the toxic staining dyes currently in use.

#### 2.4.2. Detection of Drugs

Kim et al. [78] developed a fluorescence-quenching system suitable for the detection of methamphetamine precursors, which relies on C-dots synthesised via hydrothermal treatment of a mixture of urea and citric acid (molar ratio 10:1). The C-dots have an average size of 2 nm and possess an amorphous core, while amine groups and carboxylic acid groups are present on their surface. Two methamphetamine precursors, phenylpropane-1,2-diol (PAC-diol) and phenylpropan-2-one (P2P), were found to quench the fluorescence of aqueous suspensions of these C-dots in a manner that plots linearly with the concentration of the precursors. Likewise, C-dots immobilised on a glass coverslip were able to detect the illicit precursors present in an aqueous dispersion. PAC-diol had a more pronounced quenching effect due to its two OH groups being able to bind more strongly to the C-dots’ surface compared to P2P, which bears a single carbonyl group. The related compound amphetamine sulphate quenched fluorescence to a small extent, while the common diluents paracetamol, aspirin, caffeine, glucose and sodium chloride were found to have no effect on fluorescence. In addition, substances likely to be found in illicit drug labs, namely 4-aminobenzoic acid, 4-aminophenol, aniline, benzoic acid, benzyl alcohol, hydroquinone, 4-hydroxybenzoic acid and 4-methoxyphenol had no impact on the C-dots’ fluorescence, suggesting that this system carries promise as a highly selective sensing device for crime scenes. 

**Figure 17:**
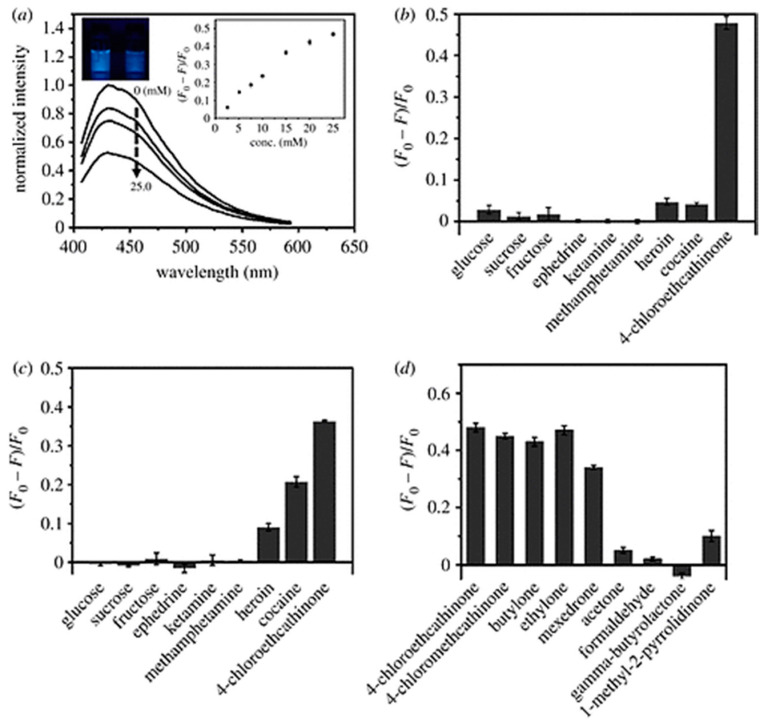
Fluorescence quenching of C-dots in aqueous suspension: (**a**) variation in fluorescence quenching at pH 11 by 4-chloroethcathinone concentration; (**b**) fluorescence quenching by a range of compounds at pH 11; (**c**) florescence quenching by the same compounds at pH 7; in (**b**) and (**c**), concentrations of glucose, sucrose, fructose, ephedrine, methamphetamine and 4-chloroethcathinone are 25.0 mM and the others are saturated; (**d**) fluorescence quenching by a range of ketones (all concentrations 25.0 mM), showing significant quenching only by those which are π-conjugated. Reproduced with permission from reference [79], Royal Society Open Science; published by The Royal Society Publishing, 2019.

Yen et al. [79] developed a system for selectively detecting and quantifying cathinones, with C-dots either in aqueous suspension or coated onto paper. The graphitic C-dots, with an average diameter of 4.4 nm, were derived hydrothermally from L-arginine and bore a range of O- and N-containing surface functional groups. While heroin and cocaine also somewhat reduced the C-dots’ fluorescence at pH 7 (Figure 17c), at pH 11, only the cathinones did so (Figure 17b,d), as heroin and cocaine are only slightly soluble at this pH. Cathinones contain π-conjugated keto groups (heroin and cocaine contain π-conjugated ester groups), which were considered to be responsible for the fluorescence quenching, given that non-π-conjugated ketones had little or no effect on the C-dots’ fluorescence (Figure 17d). The detection limit for 4-chloroethcathinone at pH 11 was found to be 1.73 mM (0.43 mg/mL). 

**Figure 18:**
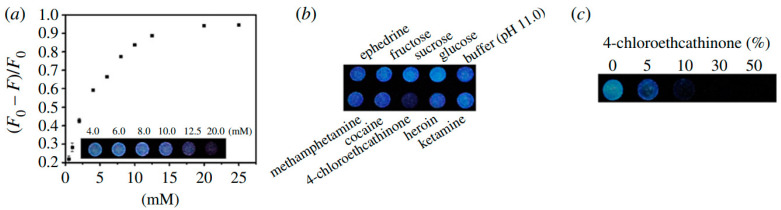
Quenching of fluorescence of C-dot-impregnated paper at pH 11, under a 254 nm portable light: (**a**) by varying concentrations of 4-chloroethcathinone; (**b**) by a range of related compounds and common diluents (cocaine, heroin and ketamine are at saturation, other compounds at 25 mM), showing clear selectivity for 4-chloroethcathinone; (**c**) at different *w*/*w*% in the presence of glucose. Reproduced with permission from reference [79], Royal Society Open Science; published by The Royal Society Publishing, 2019.

A more practicable setup for the detection of the same compound was demonstrated using C-dot-impregnated paper, a portable UV lamp (wavelength 254 nm) and a smartphone camera. The fluorescence of the paper decreased linearly as a function of the concentration 4-chloroethcathinone within the range 0.5–10.0 mM (Figure 18a), yielding a detection limit of 0.14 mM (0.03 mg/mL). Moreover, this setup is highly selective towards this compound (Figure 18b) and the emissive signal is not masked in the presence of high levels of glucose (Figure 18c). Finally, the C-dot-impregnated paper was used to detect 4-chloroethcathinone in urine, showing a linear response within the concentration range 2000–12,500 ng mL^−1^, with a detection limit of 1300 ng mL^−1^.

#### 2.4.3. Detection of Explosive Compounds

**Figure 19:**
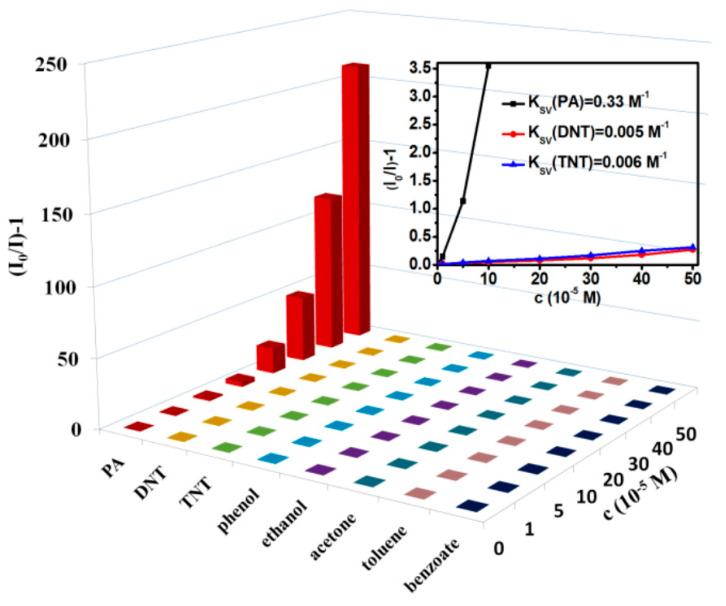
Quenching effects of a range of compounds in aqueous dispersions of C-dots at a range of concentrations, showing high selectivity for PA. Reproduced from reference [80] with permission from The Royal Society of Chemistry.

A number of groups have developed C-dots as sensitive and selective fluorescence-quenching-based detectors for explosive aromatic nitro compounds such as picric acid (PA). To that end, Niu et al. [80] used microwave treatment of equal masses (3 g each) of citric acid and urea to synthesise amorphous C-dots with diameters of 4–6 nm, bearing surface carbonyl and amine groups. Addition of PA to a C-dot dispersion induced pronounced PL quenching, yielding a detection limit of approximately 1 μM. In contrast, no PL quenching was observed in the presence of the structurally similar molecules 2,4-dinitrotoluene (DNT) and 2,4,6-trinitrotoluene (TNT) or on addition of a range of common interferents (Figure 19). In a related detection system, C-dot-impregnated paper was exposed to the vapour of DNT, TNT and PA in sealed vessels for 600 s, and the corresponding PL spectra were recorded immediately upon opening of the vessel. In this case, PL quenching was 77% for DNT and 80.3% for TNT, but only 49.8% for PA due to its low vapour pressure. 

Sun et al. [81] demonstrated that pyrolysis of ammonium citrate dibasic in a domestic microwave resulted in C-dots with a size distribution of 5.5 ± 1.5 nm, which were found to have a highly disordered structure. The product, after centrifugation for the removal of large aggregates, was a mixture of N-doped C-dots, citric acid and low-molecular-weight oligomers which was used without further purification. The fluorescence of the C-dots was systematically quenched upon the addition of increasing amounts of PA, with a calculated detection limit of 0.25 μM, while similar nitroaromatic explosives evoked no significant response.

The high selectivity of the C-dots for PA over the other nitro compounds has been attributed to its LUMO lying lower than that of the other compounds, along with the electron-deficient nature of nitro compounds, which gives rise to PL quenching due to PET. Similar levels of sensitivity to and selectivity for PA have also been reported by Siddique et al. [82], who employed C-dots, derived by ultrasonication of a mixture of dextrose and HCl, which had a range of O-containing surface functional groups but no nitrogen.

**Figure 20:**
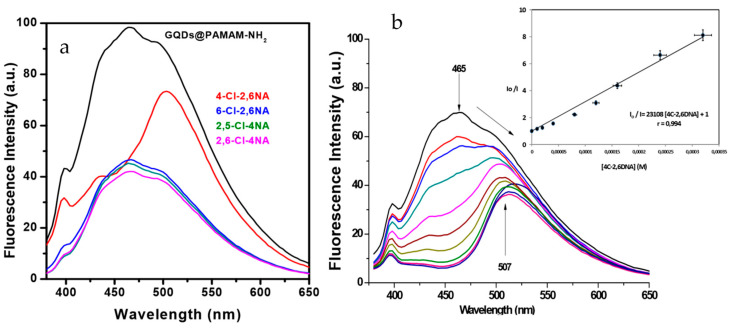
(**a**) Fluorescence quenching of PAMAM-NH_2_-capped C-dots in the presence of 4-Cl-2,6-DNA and related compounds (8 × 10^−5^ M); (**b**) fluorescence spectra of PAMAM-NH_2_-capped C-dots with various concentrations of 4-Cl-2,6-DNA: (i) 0 M, (ii) 1 × 10^−5^ M, (iii) 2 × 10^−5^ M, (iv) 4 × 10^−5^ M, (v) 8 × 10^−5^ M, (vi) 1.2 × 10^−4^ M, (vii) 1.6 × 10^−4^ M, (viii) 2.4 × 10^−4^ M, (ix) 3.2 × 10^−4^ M and (×) 6 × 10^−4^ M, with inset Stern–Volmer plot. Reproduced with permission from reference [83], Carbon; published by Elsevier Ltd., 2016.

Campos et al. [83] used a multistep oxidation of activated carbon to prepare C-dots that were subsequently functionalised with poly(amidoamine) (PAMAM-NH_2_) dendrimer. While several related compounds caused marked fluorescence quenching of the C-dots’ 465 nm fluorescence, only the explosive nitrocompound 4-chloro-2,6-dinitroaniline (4-Cl-2,6-DNA) led to the emergence of an additional emission band at 507 nm alongside the quenching of the 465 nm emission (Figure 20a). The ratio of fluorescence at these two wavelengths was found to plot linearly with the concentration of 4-Cl-2,6-DNA in the range 1.0 × 10^−5^ to 6.0 × 10^−4^ M (Figure 20b).

**Figure 21:**
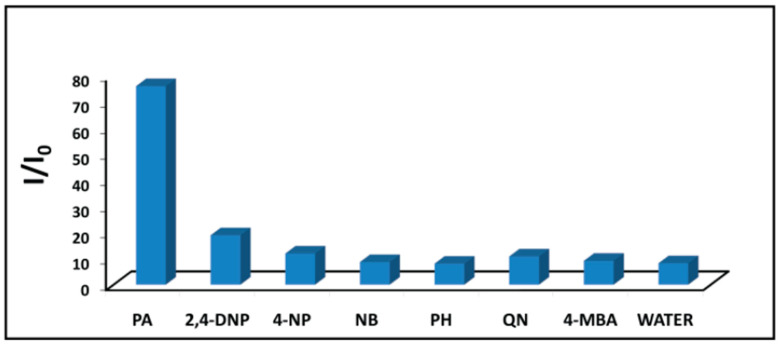
Increases in conductivity of the C-dot-PPy film in the presence of 2.0 μL 1.0 mM aqueous solution of: picric acid (PA); 2,4-dinitrophenol (2.4 DNP); 4-nitrophenol (4-NP); nitrobenzene (NB); phenol (PH); 1,4-benzoquinone (QN); 4-methoxybenzoic acid (4-MBA); water (used as a reference). The voltage was set to +5 V. Reprinted with permission from reference [84]. Copyright (2016) American Chemical Society.

Pal et al. used a C-dot-polypyrrole (PPy) composite film as a conductivity-based sensing system for PA [84] in which pyrrole was polymerised in the presence of C-dots derived from citric acid and ethylene diamine. TEM imaging of the resulting revealed that smaller C-dots, with an average diameter of 4.5 ± 2 nm, were incorporated into the PPy matrix, while larger C-dots were individually coated with PPy. Selected area electron diffraction (SAED) confirmed that the material was semicrystalline, while current-versus-voltage (I-V) plots revealed that the film exhibited metal-like conductivity attributed to networks of C = C bonds. The conductivity of the composite film reached a maximum of 2.60 mS m^−1^ compared to 0.23 mS m^−1^ for PPy. The conductivity of the nanocomposite was significantly increased in the presence of aqueous PA, while related compounds induced only minor effects (Figure 21). The detection limit against PA was found to be 1.40 × 10^−7^ M. The detection limit for PA incorporated into soil samples was 5.7 ng mg^−1^.

#### 2.4.4. Detection of Heavy Metals and Pesticides

**Figure 22:**
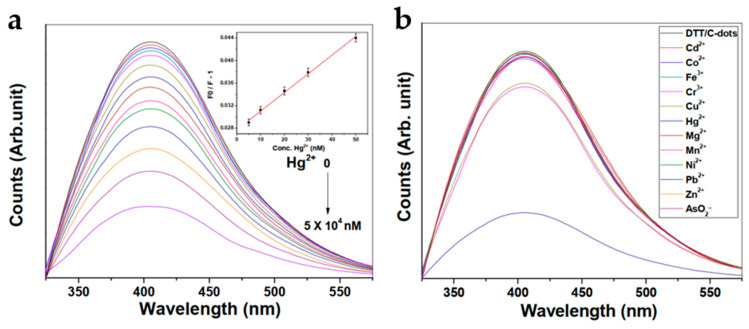
(**a**) Fluorescence quenching by Hg^2+^ ions at various concentrations, with inset Stern–Volmer plot used to determine detection limit; (**b**) fluorescence quenching capacity of a range of ions, showing strong selectivity for Hg^2+^. Reproduced from reference [85] with permission from The Royal Society of Chemistry.

Gupta et al. [85] synthesised PEGylated N-doped C-dots through microwave heating of chitosan gel in the presence of PEG followed by dithiothreitol (DTT) functionalisation. AFM and TEM suggested that the unfunctionalised C-dots had an average diameter of 8 nm and their surfaces possessed a range of O- and N-containing functional groups, although N accounted for only 2.46% of the overall mass. DTT binding was confirmed by XPS and FTIR, as was the presence of DTT’s free SH groups. The C-dots were water-soluble and stable at high salt concentrations, exhibiting the strongest fluorescence at physiological pH. Fluorescence was markedly quenched in the presence of Hg^2+^ ions (Figure 22a), while a range of other ions left the emission intensity essentially unaltered (Figure 22b). Unfunctionalised PEGylated C-dots had a detection limit of 6.8 nM against Hg^2+^ in distilled water, while DTT-functionalised C-dots could detect levels as low as 18 pM in distilled water, 45 pM in filtered (0.22 μm), centrifuged, spiked river water and 50 pM in spiked tap water. The significantly increased sensitivity of the functionalised C-dots has been attributed to extensive binding between Hg^2+^ and the thiol groups.

**Figure 23:**
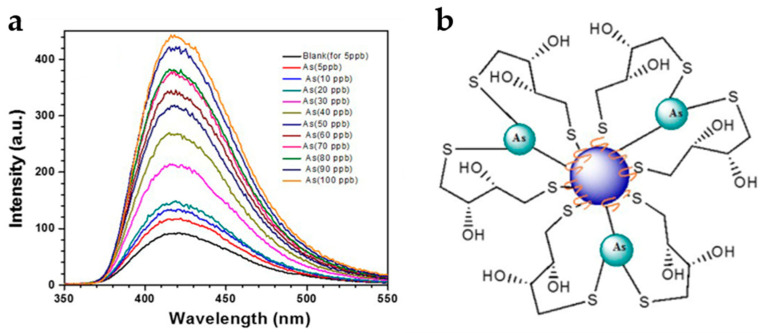
(**a**) The proposed three-point As-S binding mechanism and (**b**) fluorescence enhancement in the presence of As^3+^. Reproduced with permission from reference [86], Journal of Hazardous Materials; published by Elsevier Ltd., 2017.

D. et al. [86] synthesised C-dots via microwave pyrolysis of citric acid and cysteamine followed by DDT functionalisation. HR-TEM suggested that the size of C-dots was 4–5 nm, while SAED and XRD indicated their low crystallinity index. The C-dots had a range of O-, N- and S-containing surface functional groups, with FTIR additionally confirming binding of DTT via S-S linkages and the presence of free SH groups after functionalisation. The fluorescence intensity of the C-dots in Tris-HCl buffer was found to increase with the addition of As^3+^ ion (Figure 23a), with the limit of detection being close to 0.086 ppb. This sensing system was found to be highly selective and has been successfully used to detect As^3+^ in spiked water taken from wells, lakes and the ground. The strong selectivity of the DTT-functionalised C-dots for As^3+^ over other metal ions was attributed to the high strength of the As-S bond (379 kJ/mol), as the otherwise stronger As-O bond dissociates in water (Figure 23b).

**Figure 24:**
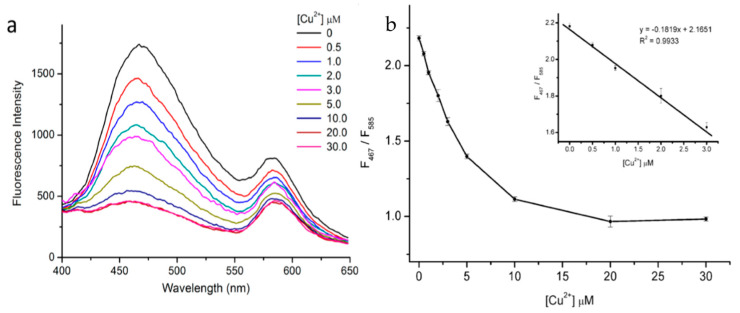
(**a**) Fluorescence spectra at differing Cu^2+^ concentrations and (**b**) plot of fluorescence intensity ratio (*F*_467_/*F*_585_) v. Cu^2+^ concentration, with inset linear fitting curve. Reprinted with permission from reference [87]. Copyright (2014) American Chemical Society.

Liu et al., developed a ratiometric, dual-emission nanosensor for Cu^2+^ ions using rhodamine-B-doped silica nanoparticles coated with C-dots, in which both fluorescent species could be excited by a single wavelength [87]. The C-dots were prepared through pyrolysis of N-(β-aminoethyl)-γ-aminopropylmethyldimethoxsilane (AEAPMS) followed by addition of citric acid. The use of AEAPMS meant that residual ethylenediamine groups on the C-dot surface could be used to capture Cu^2+^ without the need for further modification, while residual methoxysilane groups could be used for attachment to the dye-doped silica nanoparticles. The as-prepared C-dots had a diameter of 2–3 nm in ethanol, while the silica nanoparticles had a diameter of approximately 145 nm, rising to ~160 nm after the attachment of the C-dots (the attachment was confirmed by XPS). Under 360 nm illumination, the hybrid material demonstrated well-resolved dual PL emission at 467 and 585 nm, corresponding to contributions arising from C-dots and rhodamine B dye, respectively. Upon addition of Cu^2+^ (within the concentration range 0–10.0 μM), the C-dot emissive contribution decreased substantially, while the dye’s signal exhibited a minor change (Figure 24a). The ratio of fluorescence intensities at 467 and 585 nm decreased linearly with increasing Cu^2+^ concentration up to 3 × 10^−6^ M (Figure 24b). The detection limit of this system was calculated to be 35.2 nM based on a signal-to-noise ratio of 3. The fluorescence ratio in the presence of Cu^2+^ was only mildly affected by pH changes between pH 5.0 and 10.0. The system was found to be highly selective and was successfully used to quantify Cu^2+^ in spiked tap water and in biological samples.

In other work on metal ion detection, C-dots immobilised in spherical polyelectrolyte brushes were found to sensitively and selectively detect the presence of lead ions [88], while a polyaniline/C-dot nanocomposite has been developed as a fluorescent probe to detect mercury [89].

**Figure 25:**
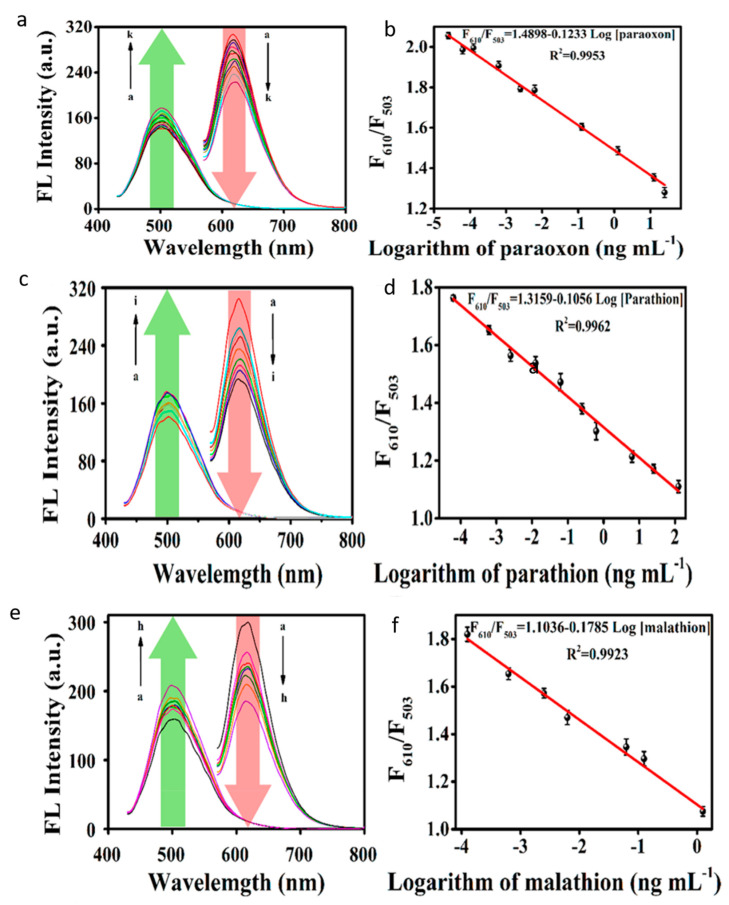
Increase in 503 nm fluorescence of polydopamine (PDA) and concomitant decrease in 610 nm fluorescence of C-dots with increasing concentration of (**a,b**) paraoxon, (**c,d**) parathion and (**e,f**) malathion, with corresponding standard curves allowing quantitation of OP levels. Reprinted with permission from reference [90]. Copyright (2020) American Chemical Society.

Li et al. [90] demonstrated an organophosphorus pesticide (OP) sensing system using red-emissive C-dots derived hydrothermally from a mixture of thiourea and citric acid. The C-dots were treated first with NAOH and then with HCl to improve their optical properties. TEM images showed a size range of 4–7 nm, while AFM suggested their height was 1.8–3.8 nm, pointing to the presence of six to twelve layers of graphene-like sheets. The graphitic nature of the C-dots was confirmed by XRD and Raman spectroscopy, while FTIR suggested a range of O-, N- and S-containing surface functional groups. The C-dots were soluble in a range of organic solvents including ethanol, DMSO, methanol and DMF and showed strong PL emission around 610 nm under 550 nm excitation. The OP ratiometric sensor relied on the polymerisation of dopamine (DA) to polydopamine (PDA) in the presence of C-dots, giving rise to a polymeric matrix that produced an emissive signal at 503 nm that quenched the PL of C-dots via PET. Upon addition of OPs (paraoxon, parathion and malathion), the PL contribution stemming from PDA was enhanced, while the contribution from C-dots weakened (Figure 25). The limits of detection for paraoxon, parathion and malathion were found to be 0.025, 0.0625 and 0.125 pg mL^−1^, respectively, lower than the allowed residue limits for these chemicals. In addition, the selectivity of the sensor system was found to be undiminished by a range of ions, amino acids and proteins found in the body, as well as a range of other pesticide families. Analysis of spiked samples of tap water, river water, soil, rice, apple and serum was carried out and confirmed the reliability of the sensing system.

Sensitive and selective C-dot-based systems for the detection of methyl parathion [91] and 2,2-dichlorovinyl dimethyl phosphate, known as dichlorvos or DDVP [92] have also been reported.

## 3. Critical Appraisal of Progress Achieved, Persistent Challenges and Future Perspectives 

### 3.1. Latent Fingerprint Enhancement

A survey of the literature indicates that research in this field is driven mainly by materials scientists, and that there is disproportionate emphasis on the photo-optical performance of the powders rather than their fingermark-detection capabilities. Obviously, a close synergy between materials scientists and forensic investigators and practitioners is critical for the advancement of this multidisciplinary and highly demanding field. A meaningful step towards this goal could be the wider adoption of experimental designs and general guidelines published by the International Fingerprint Research Group [93]. In accordance with these guidelines, it would appear that the majority of published articles present investigations that could hardly be billed as phase 1 pilot studies, given that phase 2 refers to optimisation and comparison, phase 3 to validation and phase 4 to operational evaluation and casework trials [94].

Moreover, systematic studies should be conducted to evaluate the possible health risks and short- and long-term toxicity of C-dot-based systems, and safe practices within the forensic field should be established. The nanoparticle content in powder preparations can be very low, and well-designed preparation protocols such as controlled drying coupled with the inherent tendency of C-dots to adhere strongly to diluent materials might guarantee the absence of unbound nanoparticles during dusting, though this has yet to be explored in detail. However, this reassurance will be a prerequisite for the wider engagement of the forensic community with this new class of nanomaterials. 

At the same time, recently introduced C-dot-based preparations, both powders and dispersions, should be fully assessed with respect to their capabilities in recovering fingerprints deposited on a variety of porous and non-porous surfaces, including fingermarks contaminated with body fluids or those exposed to extreme environmental elements, as well as their potential to detect DNA in lifted fingerprints. The possibility also exists that C-dot formulations applied in fingerprint development could detect metabolites and other behavioural and lifestyle indicators, as has already been shown for gold nanoparticles. 

### 3.2. Anti-counterfeiting

A growing body of literature indicates that C-dot-based nanotags for object authentication can be prepared following green protocols that require surprisingly low capital investment, providing a realistic opportunity for developing countries where counterfeit manufacturing is booming. The relatively simple and inexpensive synthesis of C-dots is an advantage in comparison to related technologies, and the significant potential of C-dot-based formulations for anti-counterfeit purposes has been clearly demonstrated, but innovative approaches to the large-scale fabrication of high-security inks and nanotags have yet to be established and a series of crucial issues need to be fully addressed before a viable manufacturing model is designed and implemented. 

The spectral response and structural stability of C-dot-based authentication patterns against humidity and adverse storage conditions, the effects of local climate and compatibility with a range of standard supporting substrates should be systematically assessed. In addition, printing techniques should be optimised to meet the requirements of each of the targeted sectors and incorporation of ink formulations currently in widespread use should be considered. Substantial input from digital engineers is required to develop technologies to capture, process and analyse the encryption images, facilitating progress towards a sustainable, automated and reliable anti-counterfeit system.

### 3.3. Molecular Sensing

The lack of standardisation strategies remains a major concern in the field of nanomaterials; this is particularly true with respect to forensic nanosensors. Unfortunately, this compromises the reliability and reproducibility of published experimental studies and prevents meaningful comparison between different studies and against established benchmarks [95]. A number of initiatives actively promote the introduction of minimum reporting requirements for nanosensors [96], including accurate and detailed synthetic protocols, comprehensive structural characterisation of the nanoparticles (elemental composition, size, shape, porosity, surface area, hydration level, zeta potential, surface functionalities, dispersity index, presence of impurities) and metrics such as analytical performance, detection limit, range of linearity, stability, repeatability and reproducibility. 

The advantages of C-dot-based sensors, in particular, are demonstrated by strong experimental evidence, but more systematic efforts are needed to realise their full potential in real-life applications. It is crucial that strategies are developed to fine-tune the elemental composition, size and crystallinity of C-dots, as well as the density of their surface functional groups, which determines their bandgap and quantum yield. Assessing the performance of C-dot nanosensors in real case scenarios and complex biofluids such as blood, urine, gastric content, spinal fluid and aqueous humour is essential in order to optimise their immunity against parasitic signals, and to eliminate false positive and false negative readings. Their optimal storage conditions should also be clearly defined and their integration into microfluidic arrays, lab-on-chip devices and automated detection system demonstrated.

## 4. Conclusions

Although far from being thoroughly understood, C-dots represent a rapidly emerging class of nano-emitters that can support the development of new and powerful tools for law enforcement and criminal justice, assisting modern societies to combat unlawful activities, terrorism and the pirate economy. C-dots’ photoluminescent properties are highly responsive to the presence of analytes such as illicit drugs, explosives, pesticides and heavy metals, allowing them to serve as sensitive nanoprobes in forensic toxicology. The colour-tuneable behaviour of C-dot-enriched powders shows significant advantages in intelligent fingerprinting, circumventing issues related to strong background interference. Finally, C-dot-based inks and polymeric formulations can generate security prints that are virtually impossible to either predict or clone, giving them the capacity to function as nanotags for object identification and authentication. The accumulated experimental evidence, mainly in the form of proof-of-concept demonstrations, is promising and sets the ground for exciting innovations in the near future that can be achieved via a collaborative approach between scientists and practitioners across a broad spectrum of disciplines.
